# Two-Year Follow-Up of *Trypanosoma brucei gambiense* Serology after Successful Treatment of Human African Trypanosomiasis: Results of Four Different Sero-Diagnostic Tests

**DOI:** 10.3390/diagnostics12020246

**Published:** 2022-01-19

**Authors:** Raquel Inocencio da Luz, Sara Tablado Alonso, Philippe Büscher, Paul Verlé, Anja De Weggheleire, Dieudonné Mumba Ngoyi, Pati Patient Pyana, Epco Hasker

**Affiliations:** 1 Unit of Mycobacterial Diseases and Neglected Tropical Diseases, Department of Public Health, Institute of Tropical Medicine, 2000 Antwerp, Belgium; stabladoalonso@itg.be (S.T.A.); pverle@itg.be (P.V.); Adeweggheleire@itg.be (A.D.W.); ehasker@itg.be (E.H.); 2 Unit of Diagnostic Parasitology, Department of Biomedical Sciences, Institute of Tropical Medicine, 2000 Antwerp, Belgium; PBuscher@itg.be; 3 Institut National de Recherce Biomédicale, Département de Parasitologie, Kinshasa 834, Congo; mumbadieudonne@yahoo.fr (D.M.N.); ppyana@yahoo.fr (P.P.P.)

**Keywords:** *Trypanosoma brucei gambiense*, sleeping sickness, serological diagnosis, follow-up

## Abstract

Gambiense human African trypanosomiasis (gHAT), also known as gambiense sleeping sickness, is a parasitic infection caused by *Trypanosoma brucei gambiense*. During the last decades, gHAT incidence has been brought to an all-time low. Newly developed serological tools and drugs for its diagnosis and treatment put the WHO goal of interruption of transmission by 2030 within reach. However, further research is needed to efficiently adapt these new advances to new control strategies. We assessed the serological evolution of cured gHAT patients over a two-year period using four different tests: the rapid diagnostic test (RDT) *HAT* Sero *K-*SeT, ELISA/*T.b. gambiense*, *Trypanosoma brucei gambiense* inhibition ELISA (iELISA), and the immune trypanolysis test. High seropositive rates were observed in all the tests, although sero-reversion rates were different in each test: ELISA/*T.b. gambiense* was the test most likely to become negative two years after treatment, whereas RDT *HAT* Sero*-K-*SeT was the least likely. iELISA and trypanolysis showed intermediate and comparable probabilities to become negative. Stage 1 patients were also noted to be more likely to become negative than Stage 2 patients in all four serological tests. Our results confirm previous findings that trypanosome-specific antibody concentrations in blood may persist for up to two years, implying that HAT control programs should continue to take the history of past HAT episodes into consideration.

## 1. Introduction

Human African trypanosomiasis (HAT) or sleeping sickness is caused by the parasites *Trypanosoma brucei gambiense* (*T.b. gambiense*) in West and Central Africa (*gambiense* HAT or gHAT) and by *T.b. rhodesiense* in East Africa (*rhodesiense* HAT or rHAT). gHAT is the chronic form and accounts for >85% of the reported HAT cases. The trypanosomes are transmitted by tsetse flies, and whereas the East African form of HAT has an animal reservoir, gHAT is assumed to be an anthroponosis. The disease occurs in two stages: an early stage (stage 1) with unspecific symptoms during the haemolymphatic phase; and a late, meningo-encephalitic stage (stage 2) with signs of central nervous system involvement when parasites have crossed the blood–brain barrier [1,2]. The disease is usually fatal if left untreated [3].

After a major peak in gHAT cases in the late 1990s, much progress has been made in recent years and the disease is now at its lowest incidence ever; globally, less than 1000 annual cases were reported in 2019 and 2020 [4]. Encouraged by these successes, WHO has set a target of elimination of transmission of gHAT by 2030 [5]. Sustained control efforts, mainly by mobile teams who screen populations at-risk with the Card Agglutination Test for Trypanosomiasis (CATT) or rapid diagnostic tests (RDTs) and who treat parasitologically confirmed patients, have led to this remarkable progress [6].

Moving forward, sustained elimination now appears to be an achievable goal as critical innovations in diagnostics and treatment near readiness for field implementation. Combined with a well performing serological test, the single-dose oral acoziborole treatment regimen promises to be such a game-changer, offering prospects of broadening treatment criteria to seropositive cases of any age without the need for confirmation [7]. Clinical research studies are still ongoing, but expected to conclude in early 2023 [8]. Such a ‘screen and treat’ strategy would overcome the lack of sensitivity of the current diagnostic confirmation methods and the diminishing expertise/resources to implement and perform the confirmation techniques on a wide and decentralized scale [9].

Serological tests are thus expected to play a critical role in the new elimination agenda. To minimize the number of missed cases, but also to avoid massive overtreatment, near-perfect sensitivity and specificity are essential requirements for these HAT serological tests. The existing serological tests mainly use the native form of variant surface glycoprotein (VSG) antigens LiTat 1.3 and/or LiTat 1.5, although in the last years, new serological tests that use recombinant antigens have been developed [10,11]. The CATT and RDTs using native antigens are the most commonly used serological screening tests at the moment. Moving forward, enzyme-linked immunosorbent assay (ELISA) formats, performed in regional or central laboratories on dried blood spot (DBS) samples collected in the field, are also expected to gain importance [12,13,14,15]. To choose the most effective and feasible serological test or algorithm of tests for the ‘screen and treat’ strategy, further research is needed, such as ‘head-to-head’ field comparisons to obtain conclusive data on specificity. Data are also lacking on the performance of serological tests, other than CATT, as a screening tool for successfully treated gHAT patients. Though probably rare, the possibility of reinfections cannot be excluded, based on the current evidence related to protective immunity post-infection and other studies that mainly point to relapse instead of reinfection [16,17,18]. Extended seropositive results post-treatment have been described for CATT and immune fluorescence assays [19,20,21,22,23,24,25], but there is no or scant information for RDTs, ELISA, and immune trypanolysis (TL), even though TL is considered the reference standard for gHAT serology.

We thus assessed the serological evolution after successful gHAT treatment, as measured by serological tests currently available or expected to become available in endemic countries in the (post)elimination context, in order to obtain a better understanding of the usefulness of serological screening tests in gHAT patients after cure.

## 2. Materials and Methods

### 2.1. Study Design and Population

Between January and May 2021, we performed a retrospective laboratory study (serological testing) using a subset of stored plasma samples from a prospective cohort study conducted in the hospital of Dipumba, Mbuji Mayi (Kasai Oriental Province, DRC), between 2005 and 2008 [26]. This study followed a cohort of 360 stage 1 and stage 2 parasitologically confirmed gHAT patients (aged 12 years or above), from diagnosis to two years post-treatment, to investigate whether follow-up of treated gHAT patients could be shortened. The patients were followed up at 3, 6, 12, 18, and 24 months post-treatment. Patients were considered cured according to WHO recommendations that were applicable at the time (for stage 1 patients: at 24 months after treatment, no parasitological evidence of relapse and ≤5 white blood cells/µL in cerebrospinal fluid; for stage 2 patients: at 24 months after treatment, no parasitological evidence of relapse and ≤20 WBC/µL in CSF). Plasma samples were collected at baseline (enrolment, before treatment (BT)), at the end of treatment (EoT), and at each follow-up visit, and shipped for analysis and storage (−80 °C) to the Institute of Tropical Medicine in Antwerp (Belgium). The cohort study was approved by the national ethics committee in DRC and the Commission of Medical Ethics of the Institute of Tropical Medicine in Belgium (Ref 04 44 1 472). Each enrolled patient provided written informed consent, including permission for secondary use of their samples for further HAT diagnostic research.

We selected, for our secondary analysis, the subset of samples (N = 1070) from HAT patients declared cured after the two-year follow-up period (n = 163) because, with current available treatments, relapse has become rare (<5%) [7,27] (Table 1).

### 2.2. Study Procedures

Before study initiation, the stored plasma samples were aliquoted in smaller volumes for each of the tests to limit freeze–thaw cycles. Four serological tests were applied on each of the 1070 plasma samples. The tests were selected based on the following criteria: availability/current field use (RDT), reference standard HAT serology (TL), or prospects of future use in peripheral laboratories (ELISA, inhibition ELISA). Bias related to result recognition patterns was minimized by having different test operators (e.g., TL performed by another operator) and sequence variation of samples across tests. All tests were performed and read by one operator, under supervision, in the WHO Collaborating Center for Research and Training on human African Trypanosomiasis Diagnosis of the Institute of Tropical Medicine in Antwerp (Belgium).

*RDT:* The *HAT Sero-K-SeT* (CORIS BioConcept, Gembloux, Belgium) is an immunochromatographic test that makes use of the purified native variant surface glycoprotein (VSG) antigens LiTat 1.3 and LiTat 1.5, combined in one test line, for the detection of antibodies against *T.b. gambiense*. The tests (Lot: 42874D2003) were performed and interpreted (overall result: negative/positive, test line color intensity scoring) according to the manufacturer’s instructions. The intensity score ranged from 1 to 12, with 1 being negative and 12 being the most intense color (considered to be indicative of high antibody titer). If the test was invalid (no control line present), it was repeated.

*ELISA/T.b. gambiense (ELISA)* was performed following the in-house protocol that has been described previously [28,29]. Briefly, half of each plate was coated with an equimolar mixture of *T.b. gambiense* LiTat 1.3 and LiTat 1.5 VSG antigens (at 2 µg/mL each), and the other half was left antigen-free. Next, the wells were blocked with a 1% skimmed milk solution to avoid non-specific protein binding, and the samples, as well as positive and negative controls (pooled plasma from gHAT patients and from endemic controls, respectively), were added in duplicate in both halves of the plate. The plates were incubated for one hour to allow antigen–antibody binding, then washed and incubated with the conjugate (goat anti-human IgG (H+L) HRP conjugated (Jackson)) for 30 min at ambient temperature. Anti-human conjugated antibodies bind to antibodies against *T.b. gambiense*, if present. Afterwards, the plates were washed again and the substrate (3,3′,5′5′-tetramethylbenzidine (TMB)) was added to each well. The reaction was stopped after 15 min of incubation by the addition of 1N sulfuric acid. The optical densities (OD) of the samples were then read at 415 nm (Multiskan RC version 6.0, Labsystems, Helsinki, Finland) and corrected by subtracting the mean OD of the antigen-free wells from the mean OD of the corresponding antigen-containing wells. To ensure standardization and minimize variation across plates, the results were expressed as a percentage positivity (PP)-value, using the OD of the weak positive control as 100%. The samples were considered positive if the PP-value was above 100.

*Trypanosoma brucei gambiense iELISA (iELISA)* is an inhibition ELISA based on the principle that binding of monoclonal antibodies to the *T.b. gambiense* VSGs LiTat 1.3 and LiTat 1.5 is inhibited by the binding of *T.b. gambiense* antibodies present in the blood of HAT patients, as both are directed to the same epitopes. The iELISA research use only (RUO) prototype (apDia, Turnhout, Belgium) was performed following the manufacturer’s instructions [Supplementary Material 5 in [30]]. Briefly, each half of the plate was coated by the manufacturer with *T.b. gambiense* antigens LiTat 1.3 and LiTat 1.5, respectively. Negative and positive controls (pooled plasma from gHAT patients and from endemic controls, respectively) were included in duplicate to both halves of the plate, along with 150 µL of a 1:20 dilution of each sample. If present in the patient’s sample, antibodies against *T.b. gambiense* will bind to the antigens coated to the plate. After one hour, the samples were replaced by conjugate solution 1 (CONJ 1 LiTat 1.3) in one half of the plate and conjugate solution 2 (CONJ 2 LiTat 1.5) in the other half; these solutions contain anti-LiTat 1.3 and anti-LiTat 1.5 HRPO-conjugated monoclonal antibodies, which specifically bind to the antigens in the plates. After 30 min, the plates were washed, and a chromogen solution was added to each well. Then, after addition of a STOP solution, the absorbance of each well was measured at 450 nm, with reference filter 600–650 nm. To calculate the results, the OD value of the negative control was considered as allowing a 100% binding of the monoclonal antibodies. A sample was considered positive if the % inhibition of either LiTat 1.3 or LiTat 1.5 was equal or greater to 30%, and negative if the % inhibition of both antigens was lower than 30% [30].

*Immune trypanolysis test (TL)* relies on live trypanosomes expressing LiTat 1.3 and LiTat 1.5 antigens and antibody-mediated complement lysis to detect antibodies specific against *T.b. gambiense.* Plasma samples and variant-specific rabbit antisera anti-LiTat 1.3 and anti-LiTat 1.5 as positive and negative controls were tested as described in [31]. Twenty five microliters of the patient’s plasma and control sera were mixed with an equal volume of guinea pig serum, which is rich in complement, and 50 µL of a suspension of 10^7^ per mL cloned trypanosomes expressing either LiTat 1.3 or LiTat 1.5. The mix was left to incubate for 90 min at ambient temperature. If LiTat 1.3 and/or LiTat 1.5-specific antibodies are present in the patient’s sample, they will bind to the parasite’s surface and the trypanosome will be killed by antibody-mediated complement lysis. After incubation, the samples were examined by contrast microscopy (×250). A sample was considered positive when over 50% of either the LiTat 1.3 or LiTat 1.5 expressing-trypanosomes were lysed [31].

### 2.3. Data Analysis

Data entry was done using Microsoft Excel (Microsoft Corporation, 2018), and analysis was done using STATA, version 17 (StataCorp. College Station, TX, USA). Descriptive statistics are presented using frequencies, proportions, and 95% confidence intervals (95% CI). For quantitative variables, mean ± standard deviation (SD) is presented for normally distributed data, otherwise the median and interquartile range (IQR) are presented.

The probability of a test becoming negative (sero-reversion) over time was evaluated using the Kaplan–Meier survival function (as done in [32]). For each time point, the hazard ratios and its corresponding 95% CI were calculated. To compare the sero-reversion probability between stage 1 and stage 2 patients, Cox regression was applied. Next, Cohen’s Kappa statistic was used to evaluate the agreement between pairs of serological tests at the different follow-up timepoints. Cohen’s Kappa coefficient was interpreted according to the Landis&Koch scale [33]: above 0.81–1 for almost perfect agreement, 0.61–0.80 for substantial, 0.41–0.60 for moderate, 0.21–0.40 for fair, 0.00–0.20 for a slight level of agreement, and <0.00 for a poor level of agreement.

Although in practical terms, all serological tests are interpreted as either positive or negative, all of them use different metrics and thresholds to measure the intensity of the signal to distinguish a positive from a negative result. In the case of the RDT, the metric is a color scale; for ELISA, it is the PP value compared with the control; for iELISA, it is the percentage of inhibition; and for TL, it is the percentage of lysis. Based on these metrics, the Kruskall Wallis rank test was used for the comparison of continuous numeric values to evaluate the intensity of the test results over time.

## 3. Results

### 3.1. Serological Reversion Rates Post-Treatment as Measured with Four Serological Tests

We obtained valid results with each serological test for the 1070 samples. The proportion of seronegative results at each timepoint is shown in Table 2. At the end of the treatment, high seropositivity rates were observed in all tests: 100% with the RDT, iELISA, and TL and 94% with ELISA. Over time, the number of seronegative results gradually increased, but reversion to negativity remained rare when measured by RDT, iELISA, or TL. At 24 months post-treatment, more than 85% of the treated patients continued to have a seropositive test result in all three tests. Sero-reversion was more frequent with the ELISA, reaching more than 24% from 12 months post-treatment onwards and 35% at two years post-treatment.

**Figure 1:**
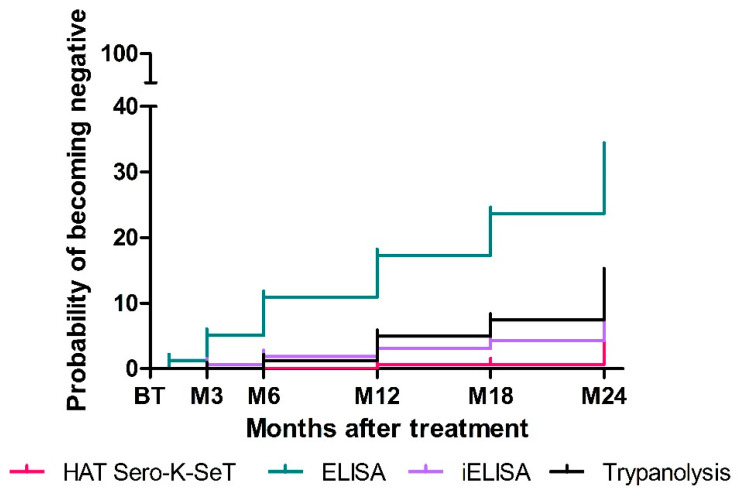
Kaplan–Meier curves displaying the sero-reversion probabilities by each serological test over a two-year follow-up period, expressed as percentages.

Based on these results, the probability of becoming negative over the 24 month follow-up period was calculated. Overall, the sero-reversion probability increases over time for all tests (Figure 1). ELISA was the serological test with the highest cumulative probability of being negative two years after treatment (42.9%; 95% CI: 34.4–52.5). The results for TL and iELISA were similar: 12% (95% CI: 6.8–20.7) and 10.4% (95% CI: 5.9–18.2), respectively. The RDT was the serological test with the lowest cumulative probability of sero-reversion (6.7%; 95% CI: 3–14.6) over the two-year follow-up period.

**Figure 2:**
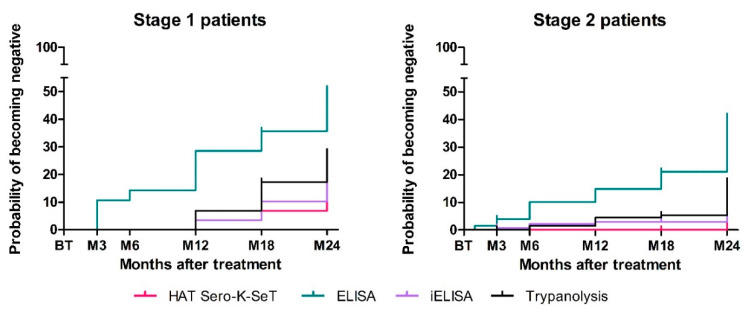
Kaplan–Meier curves displaying the sero-reversion probability of stage 1 and stage 2 patients by each serological test, expressed as percentages.

We noticed that there was a difference in the trends of sero-reversion between stage 1 and stage 2 patients. Stage 1 patients had a higher probability of becoming sero-negative over the two-year follow-up period after treatment compared with stage 2 patients in all tests (Figure 2; Table 3). ELISA remained the test most likely to become negative for both stages. Even though there was a considerable difference in the results between stage 1 (51.1%, 95% CI: 32.3–73.2) and stage 2 (41%, 95% CI: 31.8–51.6) patients, this difference was not significant (*p* = 0.18). In both TL and iELISA, the cumulative sero-reversion hazard was higher in stage 1 compared with stage 2 patients, but these differences were not significant either (*p* = 0.11 and 0.18, respectively) (Table 3). The RDT remains the test least likely to become negative, although differences can be appreciated between disease stages (10.3%, 95% CI: 2.3–37.9 for stage 1, 6%, 95% CI: 2.3–15.1 for stage 2), but again, the difference was not significant (*p* = 0.34) (Table 3).

As far as the individual hazards of becoming negative per time point, there is a significantly higher risk of becoming negative between 18 and 24 months after treatment, compared with any earlier follow-up period. In general, higher hazards were recorded for later follow-up periods (6–12, 12–18, and 18–24 months after treatment). The hazard values follow over time an incremental trend in ELISA, as can be seen in Table 3. For the RDT, the hazard of becoming negative is low and concentrated exclusively in the last follow-up period (18–24 months post-treatment), with the exception of stage 1 patients who also have a low hazard of becoming negative between 6 and 12 months after treatment. The sero-reversion hazards of iELISA and TL follow a more and similar stable path through time, with 18–24 months having the highest hazard.

### 3.2. Agreement between Tests

The proportion of concordant results and inter-test agreement (Kappa statistic) between each pair of tests was calculated at the different time points (Table 4). In general, concordance was very high, almost perfect, before and at the end of treatment (90–100%), and it decreased over time because, for some tests, a higher rate of negative results was observed. In general, the Kappa coefficient showed that agreement among the tests at different timepoints was slight to poor. As the ELISA test was the one with the most samples becoming negative over time, the concordance was systematically low and Kappa showed poor agreement from 3 months onward. When comparing RDT to iELISA, the observed percentage of concordant results was the highest over time, decreasing from 100% to 93.7%; however, the Kappa statistic declined over time. A similar trend was observed when comparing the RDT against the TL, from 3 months onwards decreasing from moderate to slight agreement. Finally, between TL and iELISA, moderate levels of agreement were observed over time with a Kappa fluctuating around the 0.46 value, while the percentage of concordance was high, decreasing slightly over time (Table 4).

### 3.3. Intensity of the Test Signals over Time

**Figure 3:**
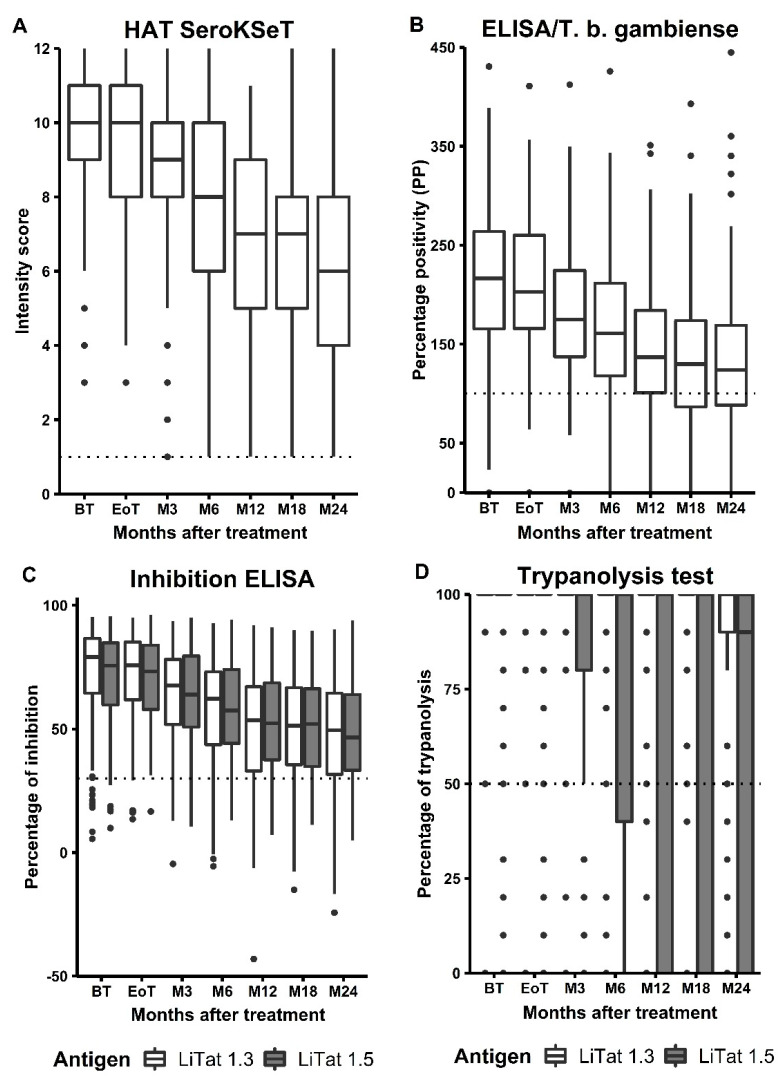
Evolution of intensity signal in the four serological tests over time. (**A**): Intensity signals of the RDT *HAT* Sero *K*-Set; (**B**): Intensity signals of the ELISA/*T.b. gambiense*; (**C**): Intensity signals of the iELISA and (**D**): Intensity signals of the immune trypanolysis test.

The evolution of the intensity of the test signals over time for the different serological tests is plotted in Figure 3. With the exception of TL, a decrease in intensity scores was observed over time. The intensity scores of RDT, ELISA, and iELISA significantly decreased over time (*p* < 0.001 for all three).

The median intensity scores of the RDT gradually decreased over time from 10.5 (IQR: 9–11; 95% CI: 10.3–10.7) to 6.5 (IQR:5–8; 95% CI: 6.1–6.9) (Table S1). A similar decreasing trend in PP values could be observed for the ELISA results. Before treatment, the median PP was 216.5 (IQR: 165.3–263.9; 95% CI: 204.3–228.7) and gradually decreased to 123.8 (IQR: 87–170; 95% CI: 113.5–134.2) after two years. In iELISA, the percentage of inhibition was evaluated for each antigen separately, and for both LiTat1.3 and LiTat1.5, a gradual decrease in the % inhibition was observed. The patterns observed here were again similar to RDT or ELISA. Before treatment, the median % inhibition was 79 (IQR: 64.5–86.7; 95% CI: 76.3–81.8) for LiTat 1.3 and 75.5 (IQR: 59.4–84.8; 95% CI: 72.4–78.7) for LiTat 1.5 and decreased to 49.5 (IQR: 31.7–64.6; 95% CI: 45.3–53.6) and 46.5 (IQR 33.2–64.2; 95% CI: 42.6–50.4), respectively. For all three tests, the overall conclusion was that the test signal intensity was significantly higher before treatment compared with two years after treatment, regardless of the metrics system used (Figure 3, Table S1).

Finally, the TL was the only test that did not show a gradual decrease in intensity. For LiTat 1.3, the percentage of lysis hardly declined over time. Two years after treatment, the median percentage of lysis was still 100, but there was higher variability with an IQR: 90–100 (95% CI: 98.7–101.3). For LiTat 1.5, the same trend was observed; however, variability started earlier, from 3 months follow-up onwards (Table S1).

## 4. Discussion

With the perspective of a ‘screen and treat’ strategy for the elimination of gHAT, we conducted this study to assess if the antibody response is still detectable in cured patients up to two years after treatment with the different serological tests that are expected to be used in gHAT control in the near future.

The results of our study indicate that the majority of cured gHAT patients continue to test positive with the latest-developed serological tests up to at least two years after treatment, despite a decreasing test signal intensity over time. For policy and practice, this means that serological test results cannot be used to decide on a possible new infection and need for treatment in cured gHAT patients. The rapid diagnostic test *HAT* Sero *K-*SeT is the test most likely to remain positive, whereas ELISA/*T.b. gambiense* is the most likely to revert to negative. The results of the iELISA and the reference test TL follow a similar trend, with about 9 out of 10 cases remaining positive at the end of the two-year follow-up. It was also noted that stage 1 patients seemed more likely to become negative in all serological tests than stage 2 patients, but the difference was not significant.

Our results confirm previous findings that trypanosome-specific antibody concentrations in blood may persist for up to two years (and beyond) after successful treatment. Lejon et al. [21] reported CATT titer results on serum samples belonging to the same collection as used in this study. Sero-reversion was much more likely in CATT, with 82% of the patients testing seronegative after two years, whereas in our study, the vast majority of serological test results, with the exception of ELISA, remained positive after two years. The difference in sero-reversion rates can be possibly explained by the fact that Lejon et al. considered CATT 1/4 dilutions as positive, so it cannot be ruled out that there would have been less negative results if undiluted samples would had been used. In field studies with a similar duration of follow-up (24–36 months post-treatment), sero-reversion rates were in the same range for CATT, but less accentuated in indirect immunofluorescence assay (IFA) in Congo Brazzaville [23,24,25]. Further downward trends in seropositivity were observed in studies that followed patients for a longer time period: CATT results reverted mostly all back to negative, while TL and/or immunofluorescence assay (IFA) results still detected antibodies over ten years after treatment [3,19,20]. Earlier results related to sero-reversion rates in stage 1 and 2 patients are conflicting, and in all three studies mentioned above, the interpretation remains inconclusive owing to a small sample size [3,19,20].

Concordance between pairs of tests remained generally high over time, except for the pairs including ELISA owing to the higher proportion of negative test results with this latter from three months of follow-up onwards. As for test agreement (measured by the Cohen’s Kappa coefficient), before and immediately after treatment, agreement was high, almost perfect. Over time, however, the number of negative tests results increases, thus resulting in a Kappa statistic that considers the expected chance agreement to be higher, thus lowering the value of the Kappa to unexpected slight and poor agreement levels [34]. In our study, this phenomenon (also referred to a ‘Kappa paradox’) can be explained by the expected chance agreement that is increased owing to the high number of positive samples in our sample set, but agreement between negative samples is mathematically very poor, while the proportion of concordant results is high. Interestingly, the Kappa coefficient of iELISA and TL in later follow-ups remained stable and with moderately high values, as these tests had a higher agreement regarding what they measured as negative. In combination with a similar hazard probability over time, and the probability of the test becoming negative being comparable to the TL, our study adds to the evidence that the epitopes reacting with antibodies in the patient sample are the same in TL as in iELISA. With its advantages such as reduced biohazard risk and increased throughput [30], the iELISA might be a good alternative for TL, but further diagnostic performance validation studies are needed.

Finally, investigating the intensity of the tests signal results over time showed that, during the two-year follow-up, no loss in % lysis was observed in TL. This was not unexpected as the analytical sensitivity of TL, i.e., the lower limit of detectable antibody concentrations, is lower than in any other antibody detection test for gHAT [3,15]. In the three other serological tests, a significant decrease in intensity was observed over the two-year follow-up. As observation stopped at this point, we are not able to firmly state or extrapolate for later timepoints, but we hypothesize that, two years after treatment and beyond, a former patient is still highly likely to present a positive result. This was demonstrated by Inocencio et al. 2021 in [35], in which a formerly treated patient presented a positive ELISA and TL result ten years after treatment. Other studies also reported TL to remain positive 12 and even up to 15 years later [3,19].

The limitations of the study are that the samples used were taken between 2005 and 2008; they have since been frozen and unfrozen several times. It is possible that some of them had suffered damage. Another element that might be considered as a limitation is that the study followed the patients for only two years; on the other hand, as such studies are rare and hard to perform, this is also the strength of this study because we could benefit from a unique collection of stored samples for which clinical data were extensively described.

Our findings have one major practical implication: as the share of RDT testing is increasing in passive screening and ELISA/iELISA relevance is expected to increase in peripheral laboratories, HAT control programs should continue to take the history of past HAT episodes into careful consideration. In the current context of very few active gHAT cases and high cure rates (>95%), a positive serology test in a cured patient is thus most likely indicative of past exposure, but not of an active infection. In the current *screen-confirm-treat* algorithm, treatment is only provided after parasitological confirmation, thus avoiding overtreatment of former HAT patients. However, if evolving towards a *screen-and-treat* strategy, this study gives an indication that successfully treated HAT patients will still test positive in most of the available serological tests even years after suffering the disease, and future treatment guidelines will need to foresee specific guidance for this subgroup of sero-suspects and decide whether one additional treatment would be reasonable. Although overtreatment of the at-risk population is expected, according to the current use of CATT and RDTs in DRC, it would not exceed 1% of the screened population. Pending further evidence on the safety and efficacy of acoziborole when used as treatment for sero-suspects, *screen and treat* could indeed become a simple yet robust enough control strategy to move towards the elimination goals.

## Figures and Tables

**Table 1:** Patient baseline characteristics and distribution of samples available for analysis (n = 1070). Stage 1 was defined by a WBC count of 0–5 cells/µL and no trypanosomes present in CSF, and the second stage was defined by a WBC count of 15 cells/µL and/or presence of trypanosomes in CSF.

	Stage 1 Patients (n = 30)	Stage 2 Patients (n = 133)
Treatment naïve/retreatment cases	30/0	53/80
Treatment		
Pentamidine	30	0
Melarsoprol	0	49
Eflornithine	0	48
Melarsoprol/nifurtimox	0	35
Melarsoprol/eflornithine	0	1
Number of patients with available follow-up samples n (%)		
Before treatment	30 (100%)	133 (100%)
End of treatment	30 (100%)	129 (97%)
3 months	29 (97%)	127 (95%)
6 months	29 (97%)	127 (95%)
12 months	27 (90%)	118 (89%)
18 months	27 (90%)	105 (79%)
24 months	28 (93%)	131 (98%)
***Total number of samples***	*200*	*870*

**Table 2:** Proportion of seronegative results (with 95% CI) by serological test and per follow-up visit.

	BT (n = 163) *	EoT (n = 159)	3M (n = 156)	6M (n = 156)	12M (n = 145)	18M (n = 132)	24M (n = 159)
*HAT Sero-K-SeT*	0 -	0 -	0.6% (0–1.8)	0.6% (0–1.8)	0.7% (0–2.1)	1.5% (0–3.6)	3.8% (0.8–6.8)
ELISA	7%	6%	9%	16%,	24%	32%	35%
	(3.1–10.9)	(2.3–9.7)	(4.5–13.5)	(10.2–21.8)	(17–31)	(24–40)	(27.6–42)
iELISA **	2.5%	0	1.9%	2.6%	6.2%	5.3%	7.5%
	(0.1–4.9)	-	(0–4)	(0.1–5.1)	(2.3–10.1)	(1.5–9.1)	(3.4–11.6)
TL **	0	0	0.6%	1.9%	7.6%	9.1%	14.5%
	-	-	(0–1.8)	(0–4)	(3.3–11.9)	(4.2–14)	(9–20)

* BT: before treatment, EoT: end of treatment, 3–24M: 3 to 24 months follow-up time point. ** For iELISA and TL, combined Litat 1.3 and 1.5 results are shown.

**Table 3:** Sero-reversion hazards (as percentages) for each timepoint and serological test, with 95% confidence intervals.

All Patients (n = 163)									
		BT	EoT	3M	6M	12M	18M	24M	Sero-Reversion Probability
RDT	Hazard	0	0	0	0	0.6	0	6.3	6.7
	95% CI	-	-	-	-	0–1.8	-	0.8–1.19	3–14.6
ELISA	Hazard	3.13	1.3	4	6.3	7.5	8.1	25.6	42.9
	95% CI	0.4–5.9	0–3.1	0.8–7.1	2.2–10.4	2.8–12.1	3.1–13.2	12.8–38.5	34.4–52.5
iELISA	Hazard	0	0	6	1.3	1.3	1.3	6.6	10.4
	95% CI	-	-	0–18	0–3	0–3	0–3.1	0.8–12.3	5.9–18.2
TL	Hazard	0	0	0	0	2.6	0.7	9.5	12
	95% CI	-	-	-	-	0.1–5	0–2	2.5–16.8	6.8–20.7
Stage 1 patients (n = 30)									
RDT	Hazard	0	0	0	0	3.5	0	7.4	10.3
	95% CI	-	-	-	-	0–10.4	-	0–21.9	2.5–37.9
ELISA	Hazard	3.4	0	11.3	4.1	18.2	10.8	23.5	51.1
	95% CI	0–10.4	-	0–24.1	0–12.1	0.4–35.9	0–25.8	0–55.9	32.3–73.2
iELISA	Hazard	0	0	0	0	3.5	7.6	8	17.4
	95% CI	-	-	-	-	0–10.4	0–18	0–23.7	6.4–42.1
TL	Hazard	0	0	3.5	0	7.4	12.5	9.1	27.8
	95% CI	-	-	0–10.4	-	0–17.7	0–26.6	0–26.9	13.6–51.4
Stage 2 patients (n = 133)									
RDT	Hazard	0	0	0	0	0	0	6.2	**6**
	95% CI	-	-	-	-	-	-	3.1–12.2	2.3–15.1
ELISA	Hazard	3	1.6	8.22.4	6.7	5.4	7.7	26	**41.1**
	95% CI	0.1–6	0–3.7	0–5.1	2.1–11.4	1.1–9.6	2.4–13	12–40	31.8–51.9
iELISA	Hazard	0	0	0.8	1.5	0.8	0	6.3	**8.9**
	95% CI	-	-	0–2.2	0–3.6	0–2.3	-	0.1–12.5	4.4–17.7
TL	Hazard	0	0	0	1.5	3.9	0.8	13.1	**17.6**
	95% CI	-	-	-	0–3.6	0.5–7.3	0–2.4	4.1–22.2	10.9–27.8

**Table 4:** Proportion of concordant results and agreement (Kappa) between tests at different timepoints.

	BT	EoT	M3	M6	M12	M18	M24
RDT vs. ELISA	92.6%	94.3%	91.7% (0.12)	84.6% (0.06)	76.6% (0.04)	69.7% (0.06)	67.9% (0.1)
RDT vs. iELISA	97.5%	100%	98.7% (0.49)	98.1% (0.39)	94.5% (0.19)	94.7% (0.2)	93.7% (0.42)
RDT vs. TL	100%	100%	100% (1)	98.7% (0.5)	93.2% (0.16)	92.5% (0.27)	84.3% (0.08)
ELISA vs. iELISA	93.9%	94.3%	92.9% (0.33)	86.5% (0.24)	82.1% (0.34)	73.4% (0.21)	70.4% (0.19)
ELISA vs. TL	92.6%	94.3%	91.7% (0.12)	84.6% (0.11)	76.5% (0.16)	75.8% (0.3)	72.3% (0.29)
iELISA vs. TL	97.5%	100%	98.7% (0.49)	96.8% (0.27)	93.2% (0.46)	93.2% (0.49)	89.3% (0.46)

## Data Availability

The data supporting the findings of this study/publication are retained at the Institute of Tropical Medicine, Antwerp, and will not be made openly accessible owing to ethical and privacy concerns. Data can, however, be made available after approval of a motivated and written request to the Institute of Tropical Medicine at ITMresearchdataaccess@itg.be.

## Supplementary Materials

The following supporting information can be downloaded at https://www.mdpi.com/article/10.3390/diagnostics12020246/s1, Table S1: median intensity scores of each serological test.
