# Supplementary material for: Two-Year Follow-Up of Trypanosoma brucei gambiense Serology after Successful Treatment of Human African Trypanosomiasis: Results of Four Different Sero-Diagnostic Tests
Source: Diagnostics (Basel). 2022 Jan 19;12(2):246. doi: 10.3390/diagnostics12020246 (PMC8871350; doi:10.3390/diagnostics12020246)
Supplement: Supplementary file 1 [file diagnostics-12-00246-s001.zip › diagnostics-1517289-supplementary.pdf]

Table S1: median intensity scores of each serological test.

RDT (color score)				ELISA (PP value)		
Follow-up	median	IQR	95%CI	median	IQR	95%CI
BT	10,5	9-11	10.3-10.7	216.5	165.3-263.9	204.3-228.7
AT	10	8.5-11	9.7-10.3	202.9	164.6-260.8	190.9-214.9
3M	9	8-10	8.7-9.3	174.7	137.2-224.8	163.6-185.7
6M	8,3	6.5-10	7.9-8.7	160.9	117.6-211.8	149.0-172.8
12M	7,5	6-9	7.1-7.9	136.8	100.6-184.1	125.9-147.8
18M	7,5	5.8-8.5	7.1-7.9	129.9	85.2-174.6	117.6-142.2
24M	6,5	5-8	6.1-6.9	123.8	87.5-170.0	113.5-134.2
**iELISA (% inhibition)**		**LiTat 1.3**			**LiTat 1.5**	
**Follow-up**	**median**	**IQR**	**95%CI**	**median**	**IQR**	**95%CI**
BT	79.0	64.5-86.7	76.3-81.8	75.5	59.4-84.8	72.4-78.7
AT	75.7	61.7-85.1	72.8-78.6	73.3	57.9-83.9	70.1-76.6
3M	67.6	51.9-78.2	64.3-70.9	63.9	50.6-79.5	60.2-67.6
6M	62.3	43.5-73.3	58.5-66.0	57.5	44.0-74.0	53.7-61.3
12M	53.5	33.1-67.1	49.1-58.0	52.3	37.5-68.7	48.2-56.4
18M	51.3	35.5-66.8	47.0-55.6	52.1	34.6-66.5	47.7-56.4
24M	49.5	31.7-64.6	45.3-53.6	46.5	33.2-64.2	42.6-50.4
**TL (% of lysis)**		**LiTat 1.3**			**LiTat 1.5**	
**Follow-up**	**median**	**IQR**	**95%CI**	**median**	**IQR**	**95%CI**
BT	100	100-100	-	100	100-100	-
AT	100	100-100	-	100	100-100	-
3M	100	100-100	-	100	80-100	97.5-102.5
6M	100	100-100	-	100	40-100	92.4-107.6
12M	100	100-100	-	100	0-100	86.9-113.1
18M	100	100-100	-	100	0-100	86.9-113.8
24M	100	90-100	98.7-101.3	90	0-100	77.5-102.5
